# The Effect of Grouper Bone Nano-Calcium (GBN) and Medium-Chain Triglyceride (MCT) Supplementation on the Ovariectomized Rats

**DOI:** 10.1155/jnme/4832594

**Published:** 2024-11-15

**Authors:** Pipin Kusumawati, Yudi Pranoto, Priyanto Triwitono, Fourier Dzar Eljabbar Latief

**Affiliations:** ^1^Department of Food and Agriculture Product Technology, Faculty of Agricultural Technology, Universitas Gadjah Mada, Yogyakarta 55281, Indonesia; ^2^Sekolah Tinggi Pariwisata Ambarrukmo, Banguntapan, Bantul, Yogyakarta 55198, Indonesia; ^3^Micro-CT Laboratory, Faculty of Mathematics and Natural Sciences, Institut Teknologi Bandung, Bandung 40132, Indonesia

**Keywords:** animal model, bioavailability, grouper bone nano-calcium, medium-chain triglyceride, ovariectomy rat

## Abstract

The objective of this research was to investigate the calcium bioavailability and the influence of substituting synthetic calcium carbonate (CaCO_3_) with grouper bone nano-calcium (GBN), and medium-chain triglyceride (MCT) with long-chain triglyceride (LCT) in the diet of ovariectomized rats maintained for 8 weeks. Twenty rats were randomly divided into four distinct groups: (1) The OX-C group: AIN-93M standard + synthetic CaCO_3_; (2) the OX-D group: AIN-93M standard + no calcium; (3) the OX-1 group: AIN-93M standard + GBN; and (4) the OX-2 group: AIN-93M with MCT as lipid source + GBN. The test parameters conducted encompassed the evaluation of the rat's body weight, levels of calcium, phosphorus, and alkaline phosphatase in rat blood serum, examination of the microstructure of rat tibiae by histomorphometry and femora bones by means of 3D micro-CT image analysis, and assessment of the strength of rat femora bones by the three-point bending. The results indicated that the GBN calcium diet groups (OX-1 and OX-2) were successful substitutes for synthetic CaCO_3_ of the OX-C group. GBN calcium diet groups have shown superiority in terms of trabeculae thickness (Tb.Th), bone volume (BV/TV), bone mineral density (BMD), and particularly in bone strength evaluations. The GBN calcium diet groups exhibited serum calcium, serum phosphorus, and alkaline phosphatase levels that were comparable to those of synthetic CaCO_3_ calcium. As the calcium-deficient group, OX-D revealed a much lower and distinct performance than other groups. This research demonstrated that MCT exhibit comparable performance to LCT; however, it did not establish that substituting LCT for MCT was superior.

## 1. Introduction

Calcium is a vital mineral for human bodies. A deficiency in calcium intake to meet the body's needs led to several conditions, such as osteopenia and osteoporosis, which were widespread in numerous nations [1]. Osteoporosis is a prevalent bone structure disorder among the elderly [2]. Several factors that initiate osteoporosis are inadequate calcium and vitamin D intake during bone development (childhood to adulthood), insufficient estrogen during menopause, passive behavior, or the long-term effects of medications. Common osteoporosis symptoms include a progressively bowed spine, decreased stature, and back discomfort [3]. After menopause, a woman's body stops producing estrogen. This results in a lack of inhibition of the osteoclast differentiation process and the initiation of bone loss induced by osteoclast formation, which defeats osteoblast formation [4]. The demography of the elderly is projected to comprise one-third of Indonesia's population by 2050, posing a significant risk of osteoporosis [5].

Numerous studies have demonstrated that the biological availability of natural calcium derived from fish bones is superior to that of synthetic calcium. According to the findings of [6, 7], the in vitro bioavailability of calcium derived from fish bones was greater than that of synthetic calcium, CaCO_3_. The findings were further validated in an in vivo investigation [8].

A recent study [7] successfully extracted the grouper bone nano-calcium (GBN) from the debris of grouper bones, with a width of 47.47 nm. Despite retaining various natural fatty acids and amino acids, GBN exhibited a calcium solubility of 26.14%, according to an in vitro gastrointestinal simulation solubility test. The objective of this research project was to assess the impact of dietary calcium supplements derived from grouper fish bones and the replacement of long-chain triglyceride (LCT) with medium-chain triglyceride (MCT) on the bone profile and strength of ovariectomized adult female rats. MCT has a shorter chain than LCT, facilitating its passive diffusion through the gastrointestinal tract and subsequent entry into the bloodstream via albumin attachment [9] and improving calcium absorption [10, 11].

MCT are extensively utilized for many applications in the healthcare industry. Research studies have shown that consuming MCT supplements can reduce the intensity of seizures in individuals with epilepsy [12–14]. Adding whey protein and MCTs in cancer patients undergoing chemotherapy diet could effectively reduce damage to the digestive tract, thereby decreasing diarrhea and weight loss incidences [15]. Previous research [16] also indicated that MCT had the ability to promote cells generating mitochondria (mitochondrial biogenesis), hence reducing the impact of certain aging and neurodegenerative disorders, such as Alzheimer's and Parkinson's diseases.

Rats were utilized as an animal model in this investigation to depict the menopausal state in elderly women. To date, no experimental study has sought to evaluate the effect of ingesting MCTs to a normal calcium diet, a low calcium diet, or a diet with supplementary calcium derived from fishbone on calcium absorption in laboratory animals, especially in ovariectomized adult female rats as animal models of menopausal state in elderly women.

## 2. Materials and Methods

### 2.1. Materials

The rat diet utilized in this investigation consisted of AIN 93M-MX, a calcium-deficient mineral combination manufactured by Dyets in Pennsylvania, USA. The two different forms of calcium in the diets were: (1) synthetic CaCO_3_ procured online (Shuren Kecuang Food Additive Co, China) and (2) GBN, a product of previous research [7]. Two sources of fat in the diet were (1) MCT (MCT-Max, Indonesia) and (2) LCT (Mazola Soya Bean Oil, Indonesia). The levels of ALP, calcium, and phosphorus in rat blood serum were determined using the Diasys Diagnostic System (Heidelberg, Germany).

### 2.2. Methods

#### 2.2.1. Diet Preparation and Animals

Twenty healthy adult female Sprague Dawley rats (12 weeks; 170–180 g) were utilized in the experiment. The standard diet utilized in this study was the AIN-93M diet designed for mature rats, which was formulated as in [17].

The four formulations of diets used in this research were: (1) The OX-C group: AIN-93M standard + synthetic CaCO_3_; (2) the OX-D group: AIN-93M standard + no calcium; (3) the OX-1 group: AIN-93M standard + GBN; (4) the OX-2 group: AIN-93M with MCT as lipid source + GBN. The composition of each rat's diet group may be observed in Table 1. The diets were prepared every 2 weeks and kept refrigerated at 4°C. Rats were fed with 10% BW each morning. Free-distilled water was available *ad libitum*. The conduct of the animal experiment was approved by the Medical and Health Research Ethics Committee (MHREC), Faculty of Medicine, Public Health, and Nursing, Universitas Gadjah Mada (Ref Number KE/FK/1250/EC/2023).

According to a study finding in [7], GBN had 30.73% calcium, while CaCO_3_ had 45.19% calcium (a more detailed comparison of the proximate composition between GBN and CaCO_3_ can be seen in Table 1 in the supporting files). According to the international standard formulation for the AIN-93M diet, the diet should contain 40.04% calcium content in the form of CaCO_3_, specifically in 35 g of mineral mix. Thus, the isocalcium diets were given in order to maintain the calcium percentage consistent between the two types of calcium diet. The weight conversion was carried out for each diet group to achieve a diet with an equivalent nutrient composition to 35 g of mineral mix. Specifically, the OX-C diet required 11.025 g of CaCO_3_ and 23.975 g of calcium-deficient mineral mix. On the other hand, OX-1 and OX-2 required 15.365 g of GBN and 19.638 g of calcium-deficient mineral mix. The weight of the fat supply (either LCT or MCT) provided to the AIN-93M standard diet, specifically 40 g, with an equal amount of fat (isofat).

#### 2.2.2. The Acclimatization Period, Ovariectomy, and Maintenance

The acclimatization period was set out for 7 days. All rats were fed with the AIN-93M standard diet. All rats were administered ketamine-xylazine (60 mg/kg BW) to induce anesthesia on the eighth day. Subsequently, the ovariectomy was conducted in order to induce the menopausal phase in the rats, similar to the method taken by [2]. Twenty rats were randomly divided into four groups, with the average weight of mice in one group around 175 g. Rats were weighed every week.

#### 2.2.3. Blood Serum Analysis

The rat's blood was then collected for analysis of serum calcium (Calcium AS FS), serum phosphorus (phosphate FS), and serum ALP (ALP IFOC FS) using the Diasys Diagnostic System (Holzheim, Germany) in the 0, 4, and 8 weeks. The outcomes of the serum and reagent reactions will be calibrated using a spectrophotometer.

#### 2.2.4. Histomorphometry of Tibiae

After eight weeks of maintenance, all rats were weighed, their blood samples were collected, and they were sacrificed with ketamine-xylazine (100 mg/kg BW). The tibiae and femora bones of rats were obtained and subsequently devoid of any residual tissue. Tibiae bones were analyzed for their histomorphometry using HE staining by [18].

#### 2.2.5. Physical Measurement and Mechanical Strength of Femora

The cleaned femora were dried in a cabinet dryer (50°C; 2 h). After determining the length, diameter, and weight of the femora of the rats, they were separated into two groups. The mechanical strength of the right femora was evaluated utilizing a universal testing machine (UTM) (Zwick Z0.5, Ulm, Germany) in accordance with the three-bending test by [19]. The left femora were utilized for microcomputed tomography (μCT) assay.

#### 2.2.6. μCT Assay

The μCT assay on the femora was done using Bruker μCT SkyScan 1173 (Köntich, Belgium) following the methodology described by [20]. The scans were performed at a voltage of 80 kV, a current of 100 μA, and a rotation step of 0.2°. The detector was set up at an exposure time of 650 ms and a frame averaging of 4, which produced a set of projection images with an isotropic pixel size of 15 μm. Reconstruction of the projection images was done using NRecon software (v 1.7.3.1, Bruker, Köntich, Belgium) utilizing the GPUReconServer (engine version 1.7.3, Bruker, Köntich, Belgium), which produced a set of reconstructed images in an 8 bit bmp format.

The reconstructed images were pre-processed (re-oriented and 3D cropped) using DataViewer (v.1.6.0.0 64 bit, Bruker, Köntich, Belgium) and were analyzed further using CT-Analyzer (CTAn, v. 1.20.8.0 + 64 bit, Bruker, Köntich, Belgium) software. The fraction of trabeculae BV (BV/TV; %), trabecular thickness (Tb·Th; mm), trabecular number (Tb·N; mm^−1^), trabecular separation (Tb·Sp; mm), and BMD (using grayscale index/GI) were determined and analyzed.

**Figure 1:**
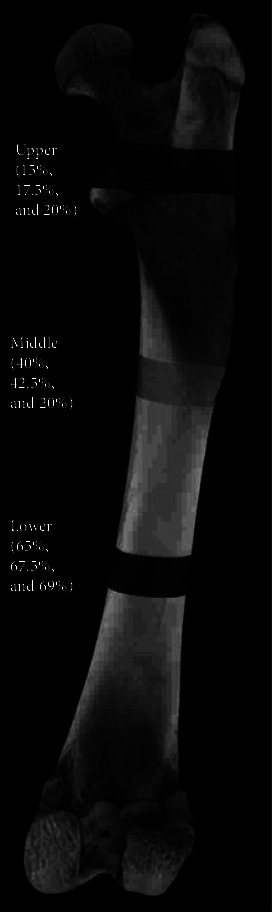
In microcomputed tomography (*μ*CT) analysis, the femora bone was divided into a planar slice and upper, middle, and lower volumes of interest (VOI).

Analysis was conducted on planar slices of 15%, 17.5%, and 20% with a volume of interest (VOI) of 15%–20% at the upper location; 40%, 42.5%, and 45% with VOI values of 40%–45% at the middle location; and 64%, 66.5%, and 69% with VOI values of 64%–69% at the lower location, in accordance with the methodology outlined by [19], assuming the overall length of the femora to be 100%.  Figure 1 provides an overview of the μCT analysis position.

### 2.3. The Statistical Analysis

All data were analyzed utilizing a one-factor Completely Randomized Design (CRD). This design comprised four diet groups with five replicates. Diversity was assessed using a one-way analysis of variance (ANOVA); when the results revealed a statistically significant difference, the Duncan Multiple Range Test (DMRT) was conducted. The statistical analysis was carried out using SPSS software (ver 26; IBM SPSS, USA), as described by [21].

## 3. Results

### 3.1. Rat's Body Weight

Body weight was evaluated weekly, before acclimatization, after acclimatization, and post ovariectomy. Data record indicated a consistent body weight gain in rats across all diet groups. However, it is interesting to note that the body weight gain of the OX-D group, which represents the calcium-deficient group, exhibited a considerable reduction and demonstrated statistical significance compared to other treatment groups between the sixth and eighth week similar to the research in [22]. This finding demonstrated that calcium deficiency negatively impacts rat growth. In the sixth week, there was no significant difference in the weight between the OX-C group and the OX-1 and OX-2 groups. However, continuing from the seventh and eighth weeks, there was a significant difference in weight between the OX-C group and both the OX-1 and OX-2 groups. In comparison to the synthetic CaCO_3_ groups, the groups fed with GBN calcium exhibited a greater body weight (data available in Figure 1 in the supporting files).

### 3.2. Blood Serum Assay

**Figure 2:**
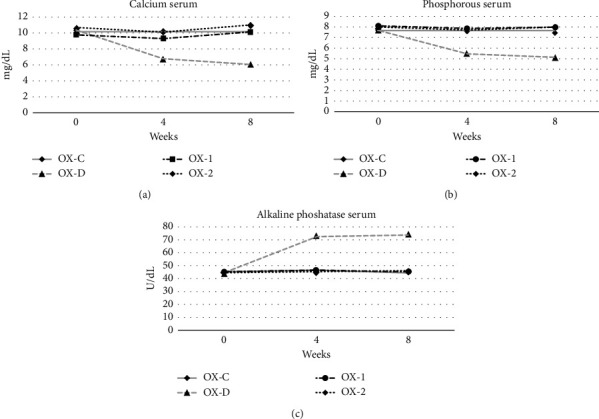
Mean plot of rat's blood serum levels over 8 weeks of observation. Diet's formula: (1) The OX-C group: AIN-93M standard + synthetic CaCO_3_. (2) The OX-D group: AIN-93M standard + no calcium; (3) the OX-1 group: AIN-93M standard + GBN; (4) the OX-2 group: AIN-93M with MCT as lipid source + GBN.

The means plot of the rats' serum calcium, phosphorus, and ALP levels for 8 weeks can be seen in Figure 2. The results for calcium and phosphorus serum levels were comparable; the serum levels of all rat diet groups were relatively stable, except for the OX-D diet group, whose levels dropped substantially in the fourth week and continued to decline until the eighth week. Evaluation of serum phosphorus and calcium levels indicated that GBN calcium source could function as an alternative to the frequently used synthetic CaCO_3_ in the diet. GBN was capable of maintaining stable serum calcium levels. Interestingly, the modified diet in the OX-D group resulted in reduced phosphorus absorption as well, in addition to calcium deficiency. This finding is in accordance with previous studies [16, 17], which revealed similar results. According to [23], calcium deficiency can cause hormonal imbalances, resulting in fatigue and laziness.

Serum ALP levels had different graphic trends than calcium and phosphorus levels. Serum ALP levels remained consistent in all diet groups for 4 and 8 weeks, except for the OX-D group. Serum ALP levels in the OX-D group showed a substantial increase in week 4 and week 8. Elevated serum ALP levels indicate heightened bone turnover, as [24, 25] stated. In the present investigation, bone turnover was specifically observed in rats fed with the calcium-deficient diet (OX-D).

### 3.3. Physical Measurement and Mechanical Strength of Femora

The physical parameters of the rat's femora in each treatment group can be seen in Table 2. The weight, length, and diameter measurements of rat femora among groups showed no significant differences. However, a significant difference was observed during the femoral bone strength tests. The OX-1 and OX-2 groups, fed with GBN calcium, exhibited significant differences compared to the OX-C group (fed with synthetic CaCO_3_ diet) and the OX-D group (fed with calcium-deficient diet).

### 3.4. Histomorphometry of Tibiae

**Figure 3:**
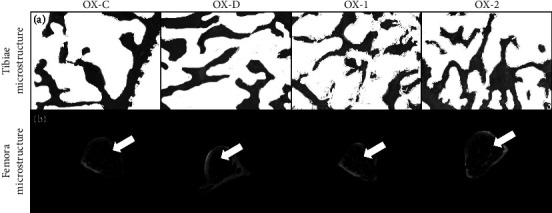
(a) Histomorphometry results of ovariectomized rat tibiae stained with HE after eight weeks of observation (40x magnification of microscope). Bone volume (BV/TV): OX-C 28.94%; OX-D 28%; OX-1 29.12%; OX-2 33.1%. (b) The microcomputed tomography (*μ*CT) image of the femora of an ovariectomized rat. The trabeculae of OX-D (calcium deficient) appear extremely sparse. The trabecular thickness of OX-1 and OX-2 seems to remain constant and comparable to that of OX-C.

The microstructure of rat tibiae and femora can be seen in Figure 3. No statistically significant differences were observed in trabecular thickness and BV at the tibial bone incision among the four diet groups (data available in Figures 2(a) and 2(b) in the supporting files). The average thickness of trabeculae in the OX-2 group was found to be equivalent to that in the OX-C group. In comparison to the OX-C group, the OX-1 and OX-2 groups exhibited a greater mean BV Figure 3(a). The group that obtained the lowest scores in both tests was the OX-D.

### 3.5. *μ*CT's Femora

The study conducted μCT assessments of Tb·Th, Tb·N, and Tb·Sp, a fraction of BV/TV, and BMD. There was no significant difference in Tb·Th outcomes between the 15% and 20% planar slice positions, but a significant difference was observed at the 17.5% position, with OX-1 and OX-2 having the highest values. The maximum Tb·Th within the 40% and 42.5% planar region of interest were found in the OX-2 group, revealing a significant difference. The planar regions of interest, with percentages of 64%, 66.5%, and 69%, had the highest trabecular thickness values in the OX-2 group, which were statistically distinct from the other groups. The results for Tb·Th VOI in the upper region were 15%–20%, in the middle region were 40%–45%, and in the lower region were 64%–69%, indicating comparable findings. The highest Tb·Th levels were seen in the OX-2 and OX-1 groups (data available in Figure 3(a) in the supporting files).

Trabecular number (Tb·N) is the quantitative measure of the number of trabeculae inside a specific area unit, expressed as the number of trabeculae per millimeter (1/mm). Tb·Sp refers to the mean distance between trabeculae [20]. The measurement of Tb·N and Tb·Sp. in rat femora bones using μCT revealed no significant variations between diets in planar slice measurements (2D) and VOI measurements (3D) (data available in Figures 3(b) and 3(c) in the supporting files).

**Figure 4:**
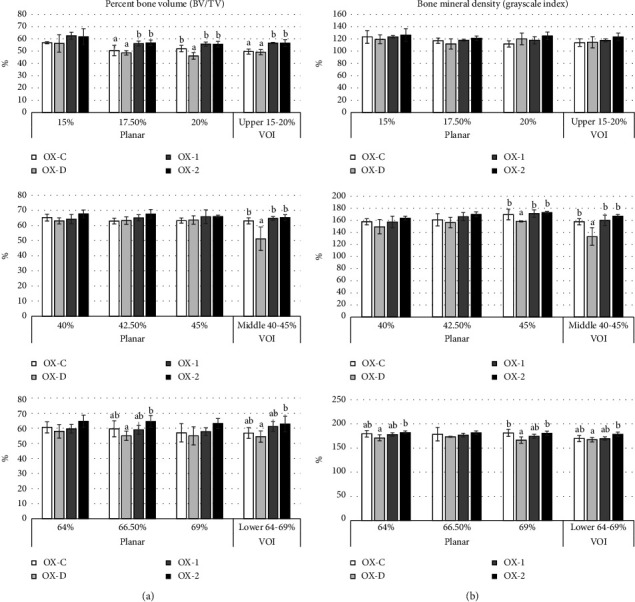
The results of percent bone volume (a) and bone mineral density (b) calculations using *μ* CT method in three test positions (upper, middle, and lower, in the planar slice position (2D) and the volume of interest (VOI) position (3D)). Data were tested using one-way ANOVA, and significant differences, followed by Duncan's post hoc test. Different lowercase letters on the same histogram indicate significant differences (*p* < 0.05). Numbers that have the same superscript letter in the same bar group were not significantly different (*p* > 0.05). Meanwhile, numbers that have different superscript letters in the same bar group show a significant difference (*p* < 0.05).

The graph of the BV value of the femora bone can be seen in Figure 4(a), and the BMD graph can be seen in Figure 4(b). The percent BV/TV results within the 15% planar slice did not significantly differ between groups. However, significant differences were observed within the 17.5% and 20% planar slices, with the OX-1 and OX-2 groups demonstrating the most favorable dietary effects. The percent BV analysis findings for planar slices within the 40%, 42.5%, and 45% regions did not reveal any statistically significant variation. The findings also discovered that BV analysis within the 64% and 69% planar slices did not exhibit statistically significant differences between treatments. However, the BV value within the 66.5% region demonstrated the highest value but was not substantially different from the OX-1 and OX-C treatments. The BV analysis results for the upper 15%–20% VOI indicated that the OX-1 and OX-2 diet groups achieved the highest scores and were significantly distinct from the remaining groups. The findings of the BV analysis for the upper and middle VOI indicated that the OX-1, OX-2, and OX-C diets had the greatest BV values, which were substantially different from the BV values of the OX-D group. The results of the BV analysis at the lower VOI indicate that the OX-2 diet had the highest BV but did not differ substantially from OX-1 and OX-C. Nevertheless, it was relatively different from OX-D.

The graph of the BMD value of the femora bone can be seen in Figure 4(b). The BMD measurements were carried out by measuring the mean of the GI in the selected VOI. No significant variations were observed between groups regarding BMD within the planar slices of 15%, 17.5%, and 20%, as well as within the upper VOI between 15% and 20%. There was no significant difference in BMD between the ROI planar of 40% and 42.5%. However, the highest BMD measurements were found within the planar slice of 45% and middle VOI at 40%–45% in the OX-1, OX-2, and OX-C groups. These three groups did not differ from each other, but they were considerably different from the OX-D group. BMD measurements within the planar region of interest (ROI) of 64% and 69% and within the lower VOI of 64%–69% revealed that the OX-2 group exhibited the highest value. The BMD in the OX-2 group was not substantially different from the OX-1 and OX-C groups but considerably different from the OX-D group. However, the planar ROI, with a percentage of 66.5%, exhibited no significant variation.

## 4. Discussion

This study aims to investigate the impact of various calcium intake sources on calcium bioavailability, the tibiae and femora bone profile, and the strength of the femora bones in postmenopausal female rats. The menopausal state was carried out by ovariectomy on the rat uterus. Menopause can be defined by a lack of estrogen production within the body. In addition to its sexual function, estrogen also inhibits the development of osteoclast cells, which are responsible for bone loss [4]. Menopausal individuals require increased calcium intake to enhance bone microstructure due to the accelerated natural bone loss caused by the lack of estrogen in the body.

Currently, calcium supplements consumed by women, particularly in the postmenopausal phase, primarily consist of CaCO_3_, produced by synthesizing inorganic substances, including limestone [26]. The preliminary study [7] successfully extracted organic calcium from grouper bone waste, demonstrating superior bioavailability in vitro compared to inorganic CaCO_3_. Another substitution made in the rat diet formula was the lipid source, with MCT substituting linoleic acid (LCT). The primary objective of this study was to investigate the potential relationship between MCT and the bioavailability of GBN.

The study analyzed the bioavailability of calcium and phosphorus in rat blood serum over 8 weeks. The results indicated that the GBN calcium group (OX-1 and OX-2) exhibited higher bioavailability than the synthetic CaCO_3_ (OX-C) group, with a significant difference that can be observed starting from the fourth week.

The microstructure testing of the femora utilizing μCT and histomorphometry methods yielded comparable results. The GBN diet groups (OX-1 and OX-2) exhibited higher values than the synthetic CaCO_3_ group (OX-C) and the highest Tb·Th levels can be observed in GBN diet groups. The outcomes of bone strength assessment utilizing the Ultimate Strength test also indicated that the GBN calcium groups (OX-1 and OX-2) were superior in terms of bone strength, with a statistically significant difference, compared to the synthetic CaCO_3_ (OX-C) group. The independent *T*-test findings indicate no significant difference between the Ultimate Strength test results of OX-1 and OX-2 groups, despite the fact that the average value of OX-2 was higher than OX-1.

The research findings demonstrated that (1) calcium deficiency can make bone microstructure appear extremely sparse on OX-D; (2) the efficacy of GBN calcium can be comparable, even higher, to the commercially available synthetic CaCO_3_. The *μ*CT images can be observed in Figure 3(b). GBN calcium has two distinct benefits: its nano size, and its rich content and variety of amino acids [7]. These attributes facilitate calcium absorption through the transcellular pathway in active absorption.

There is a limited amount of previous research investigating the correlation between MCT consumption and the level of calcium absorption in the body. The effects of consuming MCT on bone strength, whether it promotes or hinders it, is currently a subject of ongoing discussion. Prior studies conducted by [10, 27] demonstrated that premature infants with low birth weight, when provided with a diet containing sufficient amounts of calcium, phosphorus, and vitamin D, along with the inclusion of MCT, exhibited accelerated bone mineralization in approximately two-thirds of the tested infant population during the early stages of life. A study conducted in 2015 demonstrated that the inclusion of MCT in the diet of piglets resulted in enhanced growth and improved nutrient absorption [11]. Nevertheless, no studies have investigated the process by which MCTs were absorbed and how this process relates to calcium absorption. A study conducted in 2020 [28] revealed that groups treated with octanoic acid (C8), the primary constituent of MCT, demonstrated signs of bone demineralization. Nevertheless, the study pertains to the effects of a ketogenic diet over a long period of time, specifically in the absence of supplementary calcium consumption. The research findings were subsequently contradicted by a review [29] of seven more studies, which demonstrated no substantial alteration in bone mass density (BMD) among individuals who followed a ketogenic diet.

Griessen et al. found that MCT did not directly influence calcium absorption. The MCT enhances the absorption of dietary fat in the gastrointestinal tract, hence promoting the absorption of fat-soluble vitamin D. The enhanced assimilation of vitamin D is believed to enhance calcium absorption [30]. Another source asserted that bone resorption might be triggered by an inflammatory condition within the body. Inflammatory cytokines such as Interleukin (IL) 1 and 6, Tumor Necrosis Factor *α* (TNF-*α*), and Macrophage Colony-Stimulating Factor (M-CSF) promote the production of Receptor Activator of Nuclear factor-Kappa Ligand (RANKL). When RANKL binds to the RANK receptor on osteoblasts, it triggers the differentiation of osteoblast cells into osteoclasts, which is responsible for bone remodeling [31]. Additional research has indicated that inflammatory situations inside the body can lead to atypical bone mineralization [32] and may even trigger aberrant mineralization in unintended areas [33]. Research has demonstrated that children who suffer from inflammatory bowel disease (IBD) exhibited lower BMD in comparison to healthy children [34]. MCT had the ability to stimulate anti-inflammatory cytokines, specifically IL 10 and Transforming Growth Factor *β* (TGF-*β*). This led to enhanced tissue function by boosting cell mitochondrial respiration [35] and promoting cell mitochondrial biogenesis [16].

It is evident that substituting LCT to MCT in the diet yielded promising and comparable results in terms of trabecular thickness and ultimate strength. However, it was not evident whether MCT was superior to LCT in enhancing calcium absorption. The limitations of this investigation were: (1) the absence of inflammatory biomarkers in rat blood serum to verify the idea of calcium absorption facilitated by MCT; (2) the absence of Dual X-ray absorptiometry (DXA) due to the restricted availability of equipment. DXA is considered the most accurate and reliable method for evaluating BMD; (3) the rats that had undergone ovariectomy were kept for a duration of 8 weeks due to restricted resources. The rats that had undergone ovariectomy were kept for a duration of 8 weeks due to restricted resources. Therefore, we recommend that future studies should prolong the period of animal experiments to observe more pronounced alterations in study outcomes.

## 5. Conclusions

The performance of GBN calcium as a calcium source has been proven to be superior to synthetic CaCO_3_, particularly in enhancing Tb·Th, BMD, and bone strength. This research demonstrated that MCT has comparable performance to LCT but did not prove that MCT substitution was better than LCT. Based on our knowledge, no previous study has evaluated the association between MCT intake and the level of calcium absorption from fish bone debris in experimental animals. For future enhancement of the research, extending the allocated duration in growing the rats is advised to reveal more contrasting performance between the group that received GBN calcium diet along with MCT substitution (instead of LCT) and the group that just received GBN without MCT or LCT addition.

## Figures and Tables

**Table 1:** Composition of rat diet in various diet groups (in grams).

Composition (g)	OX-C∗	OX-D∗	OX-1∗	OX-2∗
Corn starch	465.692	465.692	465.692	465.692
Potassium caseinate (≥ 85% protein)	140	140	140	140
*Dextrinized* corn starch (90%–94% tetrasaccharides)	155	155	155	155
Sucrose	100	100	100	100
Soy bean oil/LCT	**40**	**40**	**40**	**0**
MCT	**0**	**0**	**0**	**40**
Fiber	50	50	50	50
CaCO_3_	**11.025**	**0**	**0**	**0**
Grouper bone nano-calcium (GBN)	**0**	**0**	**15.365**	**15.365**
Ca-deficient mineral mix	**23.975**	**35**	**19.638**	**19.638**
Vitamin mix	10	10	10	10
L-Cysteine	3	3	3	3
Choline bitartrate	2.5	2.5	2.5	2.5

Total (g)	999.92	999.92	999.92	999.92

*Note:* Numbers in bold indicate differences in composition between one feed group and another.

^∗^Diet group information: (1) The OX-C group: AIN-93M standard + synthetic CaCO_3_; (2) the OX-D group: AIN-93M standard + no calcium; (3) the OX-1 group: AIN-93M standard + GBN; (4) the OX-2 group: AIN-93M with MCT as lipid source + GBN.

**Table 2:** The physical parameter and ultimate strength of the rat's femora.

Diet groups	Femora physical parameters			Mechanical/ultimate strength (N)
	Length (mm)	Thickness (mm)	Weight (mg)	
OX-C	29.39 ± 0.009	2.89 ± 0.092	30.89 ± 1.016	34.47 ± 11.60^a^
OX-D	27.47 ± 0.017	2.81 ± 0.106	30.10 ± 0.915	31.02 ± 7.72^a^
OX-1	29.17 ± 0.011	2.91 ± 0.124	30.77 ± 0.336	51.82 ± 5.55^b^
OX-2	32.13 ± 0.074	2.97 ± 0.289	31.60 ± 2.094	58.54 ± 16.65^b^

*Note:* The mean ± standard deviation (*n* = 5). Based on one-way ANOVA, followed by Duncan's post hoc test. Numbers that have the same superscript letter in the same column are not significantly different (*p* > 0.05). Meanwhile, numbers that have different superscript letters in the same column show a significant difference (*p* < 0.05).

## Data Availability

All relevant data are included within the article.

## Supporting Information

Due to limited data that can be displayed in the manuscript, supporting files were provided in the author's personal repository. This data can be seen in a folder at the following open access link: https://drive.google.com/drive/folders/1DBfu1etNynIyxJqivScZPCX2ZO6gQ-fC?usp=sharing.
