# Supplementary material for: The Effect of Grouper Bone Nano-Calcium (GBN) and Medium-Chain Triglyceride (MCT) Supplementation on the Ovariectomized Rats
Source: J Nutr Metab. 2024 Nov 15;2024:4832594. doi: 10.1155/jnme/4832594 (PMC11585367; doi:10.1155/jnme/4832594)
Supplement: Supporting Information — Additional supporting information can be found online in the Supporting Information section. [file 4832594.f1.zip › 23-10-24-Supplementary Files.docx]

**Supplementary Files**

Table 1. The comparison of the nutritional value of two calcium sources (according Kusumawati et.al., 2022a)

| Nutrients (%) | Grouper Bone Nano-calcium (GBN) (For OX-1 and OX-2 groups) | Synthetic Calcium Carbonate (CaCO_3_) (For OX-C Group) |
| --- | --- | --- |
| Moisture (%) | 1.73±0.53 | 0.4±0.2 |
| Ash (%) | 87.73±0.04 | 99.6±0.2 |
| Protein (%) | 0.63±0.04 | nd |
| Fat (%) | 0.63±0.04 | nd |
| Calcium (%) | 30.73±0.32 | 45.19±0.57 |
| Phosphorous (%) | 18.37±0.32 | 0.0195±0.00 |
| Ca/P mole rasio | 1.29 | 1790.9 |


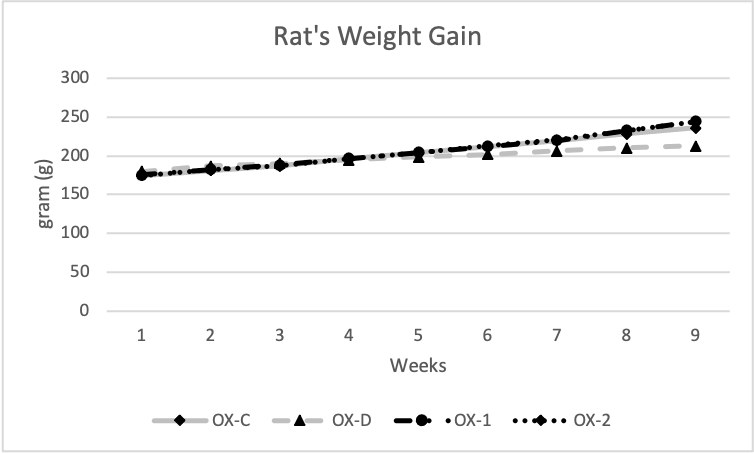


Figure 1. Rat's weight gain every week. The OX-C, OX-1, and OX-2 diet groups exhibited consistent predicted weight growth on a weekly basis. The GBN diet groups (OX-1 and OX-2) exhibited greater weight to the synthetic CaCO_3_ groups (OX-C) started at week 7 to 8. However, the OX-D diet group saw delayed weight gain. Diet's formula: (1) The OX-C group: AIN-93M standard + synthetic CaCO_3_. (2) The OX-D group: AIN-93M standard + no calcium; (3) The OX-1 group: AIN-93M standard + GBN; (4) The OX-2 group: AIN-93M with MCT as lipid source + GBN


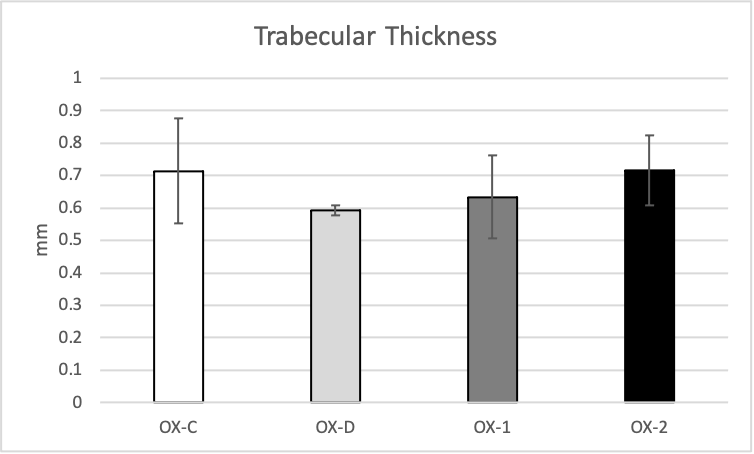

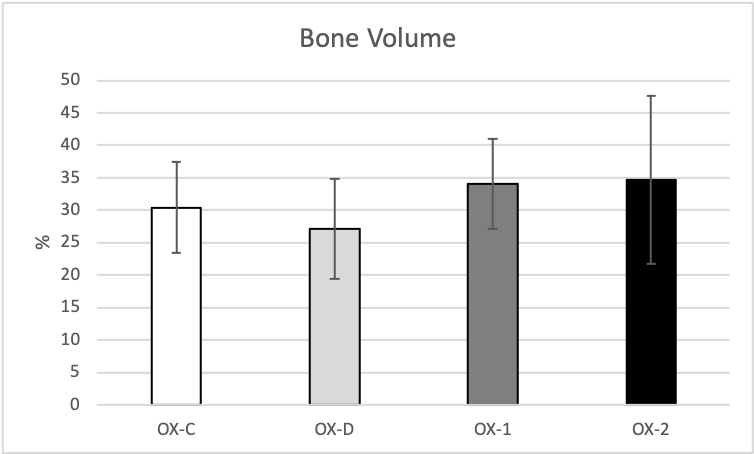


(a) (b)

Figure 2. The results of Trabeculae Thickness (a) and Bone Volume (b) using Histomorphometry method. The statistical calculations revealed that there were no significant differences seen in the data presented in the two graphs above. Diet's formula: (1) The OX-C group: AIN-93M standard + synthetic CaCO_3_. (2) The OX-D group: AIN-93M standard + no calcium; (3) The OX-1 group: AIN-93M standard + GBN; (4) The OX-2 group: AIN-93M with MCT as lipid source + GBN


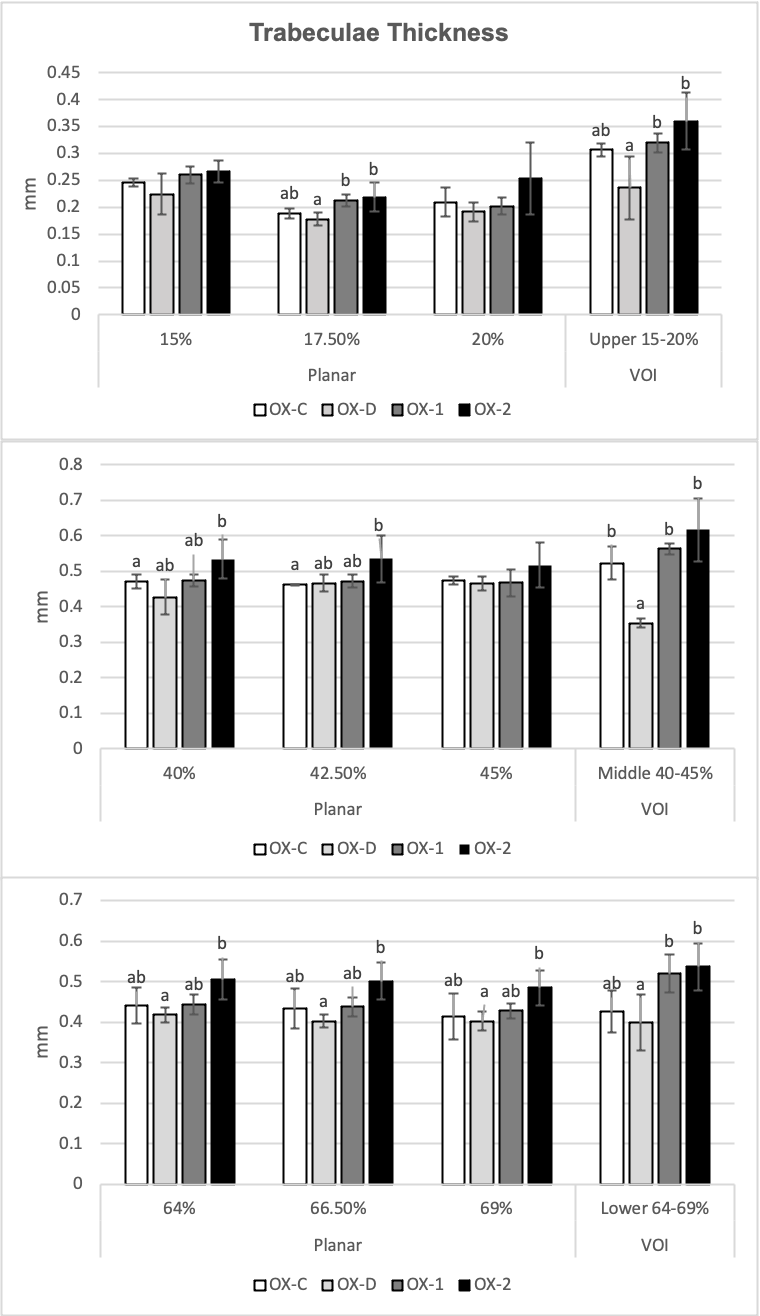

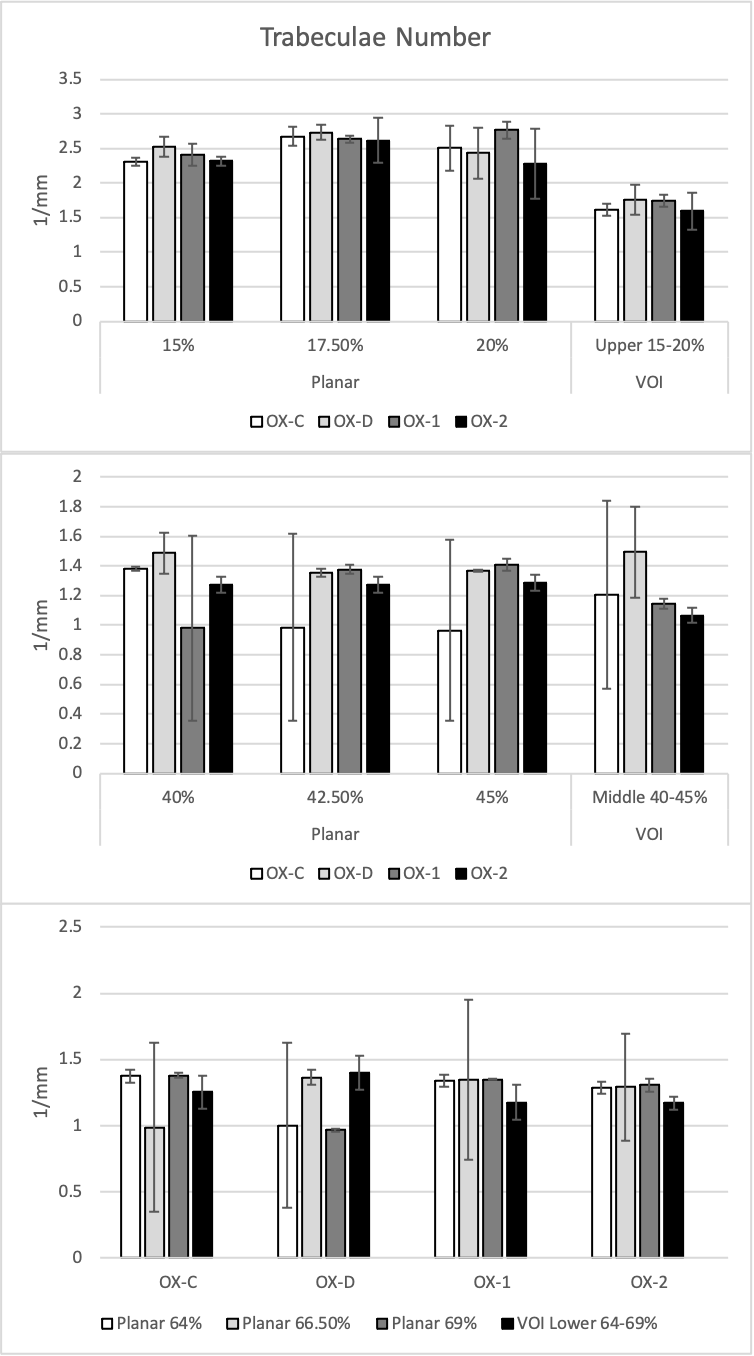


(a) (b)


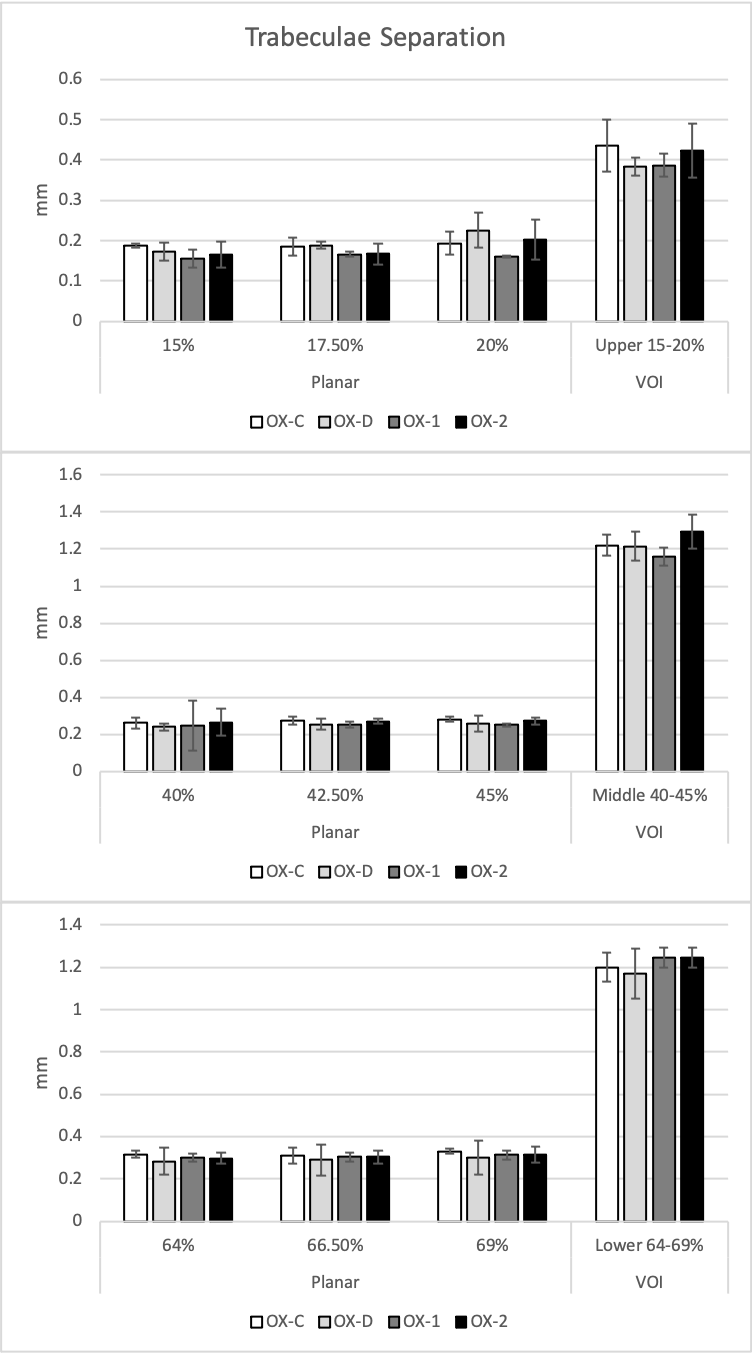


(c)

Figure 3. The results of Trabeculae Thickness (a), Trabeculae Number (b), and Trabeculae Separation (c) calculations using $\mu$CT method in three test positions (upper, middle and lower, in the planar slice position (2D) and the volume of interest (VOI) position (3D)). Data were tested using one-way ANOVA. The statistical calculations revealed that there were no significant differences seen in the data presented in the three graphs above.

Data were tested using one-way ANOVA, and significant differences followed by Duncan's post hoc test. Different lowercase letters on the same histogram indicate significant differences (P < 0.05). Numbers that have the same superscript letter in the same bar group were not significantly different (P > 0.05). Meanwhile, numbers that have different superscript letters in the same bar group show a significant difference (P < 0.05).
